# Magnetism and ε-τ Phase Transformation in MnAl-Based Nanocomposite Magnets

**DOI:** 10.3390/nano11040896

**Published:** 2021-03-31

**Authors:** Alina Daniela Crisan, Aurel Leca, Cristina Bartha, Ioan Dan, Ovidiu Crisan

**Affiliations:** 1 National Institute for Materials Physics, P.O. Box MG-7, 077125 Magurele, Romania; ad_crisan@yahoo.com (A.D.C.); lec.aurel@gmail.com (A.L.); cristinavals@yahoo.com (C.B.); 2 R & D Consulting and Services S.R.L., 023761 Bucharest, Romania; ioan_dan@rd-consultanta.ro

**Keywords:** RE-free magnets, differential scanning calorimetry, ε-τ phase transformation, magnetic properties

## Abstract

Melt spun ribbons of Mn_53_Al_45_C_2_ and Mn_52_Al_46_C_2_ have been synthesized by rapid quenching of the melt with the purpose of monitoring the ε-τ phase transformation to show technologically feasible ways to increase magnetic parameters and to illustrate the viability of these alloys as the next generation of rare earth (RE)-free magnets. By differential scanning calorimetry (DSC), activation energies and temperatures of onset of the ε-τ phase transformation were obtained. Structural analysis was performed using X-ray diffraction (XRD) and the resulting XRD patterns were quantitatively assessed using full profile Rietveld-type analysis. Appropriate annealing was performed in order to enable the ε-τ phase transformation. While hcp ε-phase was found to be predominant in the as-cast samples, after appropriate annealing, the tetragonal τ-phase, the one that furnishes the relevant magnetic response, was found to be predominant with an abundance of about 90%. The data suggested a mechanism of hcp ε-phase decomposition controlled by the segregation towards the interfacial regions, having the rate of transformation governed by antiphase boundary diffusion processes. Magnetic measurements of annealed sample Mn_53_Al_45_C_2_, consisting of predominant tetragonal τ-phase, showed high values of magnetization and increased coercivity, consistent with an energy product of about 10 MGOe, similar with previously reported magnetization measurements, providing further insight into the realization of future class of RE-free low-cost permanent magnets.

## 1. Introduction

There is a globally known quest for developing novel materials which makes little, or not at all, use of critical raw materials, as is for instance described in the Raw Materials Initiative of the European Commission [1]. Among these critical raw materials, rare earth (RE) elements are among the most depleted ones and their replacement in the making of magnetic materials is painstakingly difficult since there are not many elements that possess magnetic moment and exhibit technologically exploitable intrinsic magnetization and high enough coercive field. In search for alternative permanent magnets, which do not contain RE, the binary Mn-Al system has recently attracted a growing interest. Mn is by itself an antiferromagnet; however, by alloying with other metals, it can produce noticeable ferromagnetic features. Some binary Mn-based systems may show ferromagnetism, such as the ones including Ga, Sb, As, or Ge; however, only Mn-Al and perhaps Mn-Bi alloys have some potential in developing good coercivity associated to large enough saturation magnetization. In certain conditions, Mn-Al may show good magneto-crystalline anisotropy, good resistance to corrosion, and it is resilient and easily machinable with good mechanical strength, making it one of the most promising candidates for RE-free magnets [2,3,4,5,6].

What makes Mn-Al a good magnet is in fact its tetragonal τ-phase, that is similar to the ordered L1_0_ phases, as retrieved in FePt or CoPt magnets [7,8] with strong uniaxial magneto-crystalline anisotropy [9]. However, the formation of this phase and fine tuning of corresponding magnetic properties is a rather daunting task. The actual making of Mn-Al-based magnets proved to be difficult and showed quite low coercivity [10]. The reason is linked to the metastability of the τ-phase and that this phase forms only at quite narrow windows of composition in the binary phase diagram. τ-MnAl having tetragonal L1_0_ symmetry arises from the parent hexagonal (hcp) ε-phase, which itself is non-magnetic, at a stoichiometry of around 51–58 at.% Mn [11,12], through annealing of binary alloys, rich in manganese, at temperature values above 500–650 °C. It is known that Mn^2+^ possesses a large magnetic moment of about 5 μB/atom, but due to the antiferromagnetic alignment, this good magnetic moment cannot be exploited for in technological applications. In the binary MnAl phase diagram, at equilibrium [13], the range of 51–58 at.% Mn correspond in fact to the mixture of two phases, γ_2_-Mn_5_Al_8_ and β-Mn with diluted Al atoms. In the τ-MnAl phase that forms at almost equiatomic composition, Mn and also Al atoms occupy randomly the (0,0,0) and (½,½,½) crystallographic positions. However, as the real structure is rarely equiatomic and almost never completely ordered, some of the excess Mn atoms will accommodate few of the Al (½,½,½) crystallographic positions and consequently will align their magnetic moments antiferromagnetically, thus diminishing the overall magnetization. In some of the earlier works [14], it was found that maximum saturation magnetization was at about 51 at.% Mn and starts to decrease with increasing the Mn content. It was also found that adding C furthermore increases the magnetization at the expenses of decreasing both the Curie temperature and the coercivity of the τ-phase.

It is worth noting that the formation of τ-phase in Mn-Al depends strongly upon stoichiometry, microstructural details, as well as thermodynamic considerations. As said above, the ferromagnetic τ-phase has a tetragonal superlattice structure that is similar to other L1_0_ phases [15,16,17,18,19,20]. It has been reported [21,22] that τ-MnAl is synthesized either by the rapid quenching of ε-phase and subsequent annealing or by slowly cooling down the parent disordered ε-phase. There have been evidences that τ-MnAl may arise from hcp ε-MnAl precursor by annealing at around 500 °C, but it was reported that such method leads to certain structural instabilities of this phase [23]. In terms of phase diagram [13], L1_0_ phase has a slight tendency indeed to decompose in a mixture of solid solutions of β-(Mn) and γ_2_ (Mn_5_Al_8_) phases, above 650 °C. There are, however, means of improving this stability and preserving the structural dominance of τ-phase in Mn-Al binary alloys. Adding carbon improves the overall stability, as reported in [2] for the L1_0_ phase. Nevertheless, the majority of published results about phase transitions in MnAl leading to formation of magnetic phases with good stability were mostly theoretic until recently. Ab initio approaches have given reasons to consider that magnetic ground state of the τ-MnAl is ferromagnetic [24,25,26], having a magnetic moment of about 160 emu/g. Density functional theory approaches [27] showed that large magneto-crystalline anisotropies of about 1 MJ/m^3^ can be expected for MnAl and MnGa, together with a saturation magnetization value of about 0.8 MA/m. By means of Monte Carlo simulations, the Curie temperatures of Mn-rich off-stoichiometric compositions was found to be about 327 °C [27]. It has already been argued that the occurrence of τ-phase depends heavily on the chemical composition, Mn to Al ratio, processing routes, and subsequent annealing. It was inferred [28,29,30] that the phase transformation of Mn-Al from ε-to τ-phase is a process occurring in two steps. The first step, attributed to some thermal diffusion within the microstructure during annealing, the reorder of atoms in hcp ε-phase produces a quasi-layered ε′-phase of orthorhombic symmetry. The second step consists of the transformation of ε′-phase within a fast martensitic-type process, which provides incipiency of τ-phase formation [30]. For most of the cases irrespective of the synthesis method, a mixture of phases is obtained rather than one single phase in the Mn-Al-based alloys. In some cases [29,31,32], the microstructure was found to consist of a mixture of τ-phase and ε-phase; in other cases [33,34], an amalgam of the γ_2_, β, and τ phases was found, while in [35], another mix of structures was found: τ, β, and ε-phases. Non-equilibrium synthesis procedures such as melt spinning, rapid quenching from the melt, planetary ball milling, but also gas atomization are mostly efficient for synthesis of Mn-Al alloys [6,36]. Even if the magnetic properties of melt spun MnAl alloys [3,37] are quite similar, for ball milled samples [2], quite different properties are observed. It is considered that slight changes in the milling processing route helps obtain different grain microstructures produced by randomly induced structural defects. Only a few available works report almost single τ-phase alloys [31], this being obtained in ribbons synthesized by planar flow casting, furthermore annealed in carefully selected thermal conditions. Another way to stabilize the τ-phase formation is by careful compositional modulation, by adding for instance small amounts of carbon. It was reported [38] that τ-phase can be obtained from the melt spun alloys slowly cooled down to room temperature. Nevertheless, here the main stabilizing role was played by carbon addition rather than the cooling conditions. In a previous paper [29], the control of the cooling process at a proper rate was deemed important for acquiring a significant content of τ-phase. The need of the rapid cooling of hcp ε-phase to avoid decomposing into the equilibrium phases β-Mn and γ_2_ (Mn_5_Al_8_) [39] was also proven. Another way to obtain large amounts of τ-phase was reported [34] by using strip-cast; however, this was successfully achieved only for off-stoichiometric composition Mn_54_Al_46_ (at.%). Mitsui et al. [40] used annealing at 300 °C, under an applied magnetic field to investigate Mn-Al alloyed with Zn and also Mn-Al-C. It was found that magnetic field annealing was beneficial for the Zn-modified Mn-Al but detrimental for C-modified Mn-Al, in what concerns stabilization of τ-phase against ε-phase. Dehghan and Ebrahimi [41] have also studied influence of strain on magnetic behavior of Mn-Al-C samples, hot compressed, and they argued that higher strains lead to higher coercivity and remanence. K.P. Su et al. [42] studied Mn-Al based hard magnetic magnets with C addition, obtained as flakes by surfactant assisted ball milling. Texturing along [001] axes of τ -MnAl phase was observed, and the coercivity was found to be at around 2500 Oe even after 15 h milling. In a very recent publication, R. Kobayashi et al. [43] have shown that magnetic Mn–Al–C (τ-phase) may be produced by reactive sintering. They obtained maximal coercivities and magnetization for Mn_55_Al_45_C_2_ annealed at 1000 °C, a sample where the abundance of τ-phase was about 81%. Results have been interpreted in terms of phase stabilization by carbon addition during ε-τ transformation.

For a deeper understanding of the effects of carbon addition and of the dynamics of the ε-τ phase transitions in the MnAl-based systems, we have elaborated two alloys by means of melt spinning followed by rapid solidification of the melt, resulting in formation of thin ribbon shapes. Two different compositions have been obtained: Mn_52_Al_46_C_2_ and respectively Mn_53_Al_45_C_2_. Differential scanning calorimetry measurements have been employed to verify eventual exothermic effects during structural phase transformations as well as the corresponding activation energies. We perform structural studies by powder X-ray diffraction, a method which enables for the identification of the nature, the type, structural symmetry, and the morphology of the determined phases. The magnetic features were determined and correlated to the structural findings. These results enable us to monitor and to prove the evolution of crystalline phases in Mn_52_Al_46_C_2_ and respectively Mn_53_Al_45_C_2_ alloys.

## 2. Materials and Methods

The two alloys, Mn_52_Al_46_C_2_ and respectively Mn_53_Al_45_C_2_, have been synthesized as ribbons by rapid quenching of the melt, in a non-equilibrium synthesis process utilizing a melt spinner device from Buehler. Pre-alloys have been made starting with elemental powders of high purity, using an arc-melting technique in controlled Ar gas atmosphere of 10^−1^ Torr partial pressure, followed by re-melting 3 times for a better homogeneity of the primary alloys. In the next step, primary alloys of a total mass of 5 g were diced and then melted again in crucibles made of quartz, having 2.5 mm nozzles. The melt was purged using over-pressured Ar gas (40 kPa) on a copper wheel having 40 cm diameter. The wheel rotates at about 1600 rot/min, corresponding to a peripheral velocity of 33.5 m/s, producing long and homogeneous thin ribbons. The ribbons obtained were 2 mm wide, homogeneous, and with a thickness of about 40 μm. The elemental composition of the ribbons was measured using particle-induced X-ray emission spectroscopy (PIXE). PIXE is a nuclear experimental technique which provides accurate elemental analysis. While it is an unusual technique for solid state scientists, since it requires the use of highly energetic beam of charged particles produced in nuclear accelerators, it is quite efficient in quantitatively analyzing the stoichiometry of solid samples. In PIXE, the sample is exposed to heavy charged protons, with kinetic energy between 1 and 4 MeV, and it subsequently emits element-specific X-ray emission from the whole solid sample exposed. In comparison to the energy-dispersive X-ray (EDX) spectroscopy, PIXE has the advantage of low limits of 0.1–10 mg/kg (or 10^−5^–10^−7^) for elemental analysis, which was especially needed in our case for detecting the low Z element such as C. The elemental composition of the ribbons, as revealed by PIXE, was found to be quite close to nominal composition, within 0.5 at%. The two alloys compositions, determined from PIXE, are Mn_52.2_Al_45.6_C_2.2_ and respectively Mn_53.2_Al_45.1_C_1.7_. No contaminating oxides or oxi-hydrides were found in the samples. Calorimetry studies and thermal analysis was done with differential scanning calorimetry technique (DSC), employing the Setaram DSC 111 system, with 5 to 50 °C/min heating rates, and raising the temperature up to about 600 °C in a controlled Ar environment. The X-ray patterns were done using the Bruker D8 Advance diffractometer with Cu Kα radiation (λ = 0.154 nm). The classic θ–2θ transmission geometry was used for diffraction for angles between 20° and 90° (in 2θ). A full-profile pattern analysis was done on the obtained diffraction patterns using MAUD (Materials Analysis Using Diffraction) software. The magnetic measurements on the Mn_52_Al_46_C_2_ and respectively Mn_53_Al_45_C_2_ samples were done using a MPMS (Magnetic Properties Measurement System) from Quantum Design. Initial magnetization and hysteresis loops of samples were determined under an applied field of up to 80 kOe, which was parallel to the planar side of ribbons, at 27 °C.

## 3. Results

### 3.1. DSC Results

**Figure 1:**
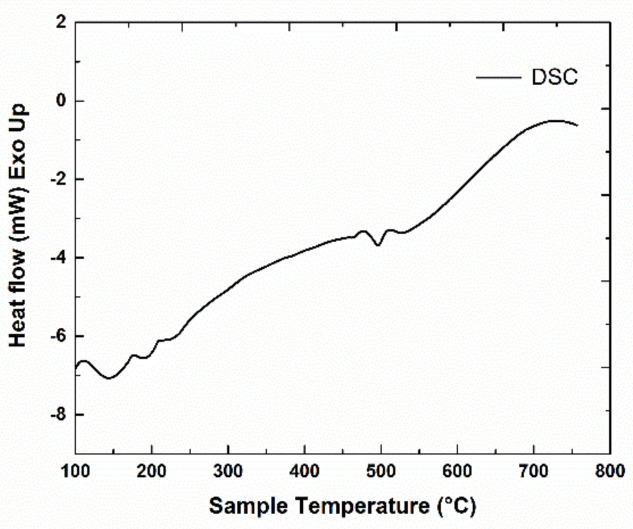
Differential scanning calorimetry (DSC) scan for Mn_53_Al_45_C_2_ sample.

**Figure 2:**
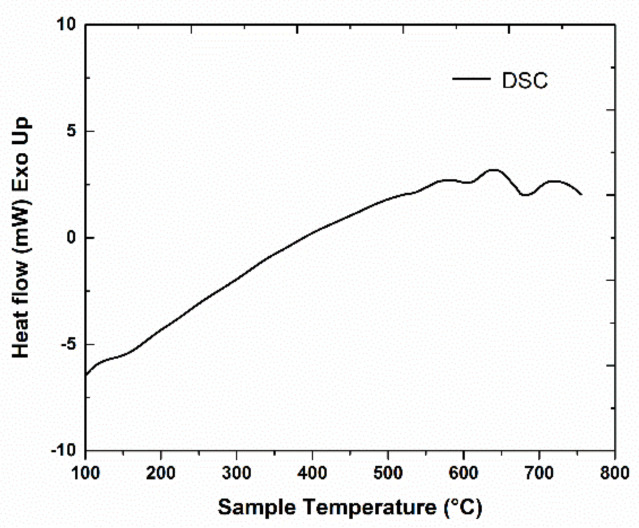
DSC scan for Mn_52_Al_46_C_2_ sample.

Regarding the phase transformation evolution with temperature in metallic alloys, one of the most promising techniques is the differential scanning calorimetry (DSC). In our alloys, DSC analysis was undertaken in order to notice and monitor thermal events or heat exchange with the environment, illustrated as exo- or endo-thermic peaks. Events that occur with temperature increase are related to intrinsic structural effects that employ change of the internal energy and are associated to structural phase transitions. The DSC scans have been performed on both the Mn-Al-C alloys. Measurements were taken from 100 °C up to 750 °C with a heating rate of 5 °C/min. The heat exchanged with the exterior environment, measured by comparison with a reference crucible heated to the same temperature, was recorded during the heating process and the data obtained for the 2 samples are presented in Figure 1 and Figure 2. Besides the constant heat flow increase with increasing temperature, few heat flow effects (exo- and endo-thermal) are noticed in both graphs. The effect attributed to the structural phase transformation leading to the formation of τ-MnAl phase was found at 504 °C for the sample Mn_53_Al_45_C_2_ (Figure 1). A similar peak was noticed also by Lu et al. [32] in Mn_55_Al_45_ at 492 °C and assigned to the occurrence of the τ-phase. An exothermic effect at 509 °C was recorded in another work [29] for Mn_0.55_Al_0.45_C_0.02_ and again assigned to the occurring of τ-phase. For the sample Mn_52_Al_46_C_2_, the exothermic effect recorded in the previous graph is postponed with about 100 °C to about 606 °C (Figure 2), this peak also being assigned to the ε-τ transformation. The delay in formation of the τ-MnAl phase is in this case most probably related to the higher activation energy induced by the increased Al content. It can be seen in the Mn-Al phase diagram [13] that the limit of formation of the τ-phase is exceeded when lowering the Mn content below 53 at.%; therefore, during the non-equilibrium heating process of the DSC study, a higher activation energy is required for triggering the formation of the τ-phase.

For more information about the ε-τ structural transformation, other relevant calorimetry parameters, such as the activation energy, may be derived from DSC thermograms, recorded for different heating rates. These parameters are important to be assessed since there is a delay of the ε-τ transformation, as seen from the different values of the transformation temperature. We infer that the retardation of the phase transition in the sample Mn_52_Al_46_C_2_ (Figure 2) with respect to that of sample Mn_53_Al_45_C_2_ (Figure 1) is due on one hand by the slight compositional changes and positioning relative to the limit of formation of τ-phase, and on the other hand is triggered by the differences in the phase structure, as revealed by the X-ray diffraction (XRD) results. We have calculated the activation energies from 5 different DSC scans recorded at various heating rates, and we obtain, for both samples, similar behavior in terms of the variation of activation energies, which increase linearly with the transformation fraction. For the estimation of activation energy, we have used the so-called model-free analysis. This analysis calculates some kinetic parameters: apparent activation energy as well as pre-exponential factor. It employs rate equation and Arrhenius law and considers that the reaction model is independent of both temperature and heating rate [44]. The average activation energies are 185 ± 7 kJ/mol for the sample Mn_53_Al_45_C_2_ and 306 ± 11 kJ/mol for the sample Mn_52_Al_46_C_2_. These average values of activation energies are comparable to the value of around 190 kJ/mol for Mn_55_Al_45_ alloys [31,32] and are also in agreement with the value reported in [37] of about 300 kJ/mol. It has been reported in many previous works [31,32,33,34,35,36,37] that the ε-τ transformation occurs gradually upon annealing. To maximize the abundance of the formed τ-phase, we have chosen an annealing temperature that is well beyond the observed DSC peaks, for permitting the system to complete the ε-τ transformation. Annealing procedures were undertaken at 700 °C for 1 h. The samples resulting after annealing have been fully characterized, structurally and magnetically, along with their as-cast counterparts.

### 3.2. XRD Results

**Figure 3:**
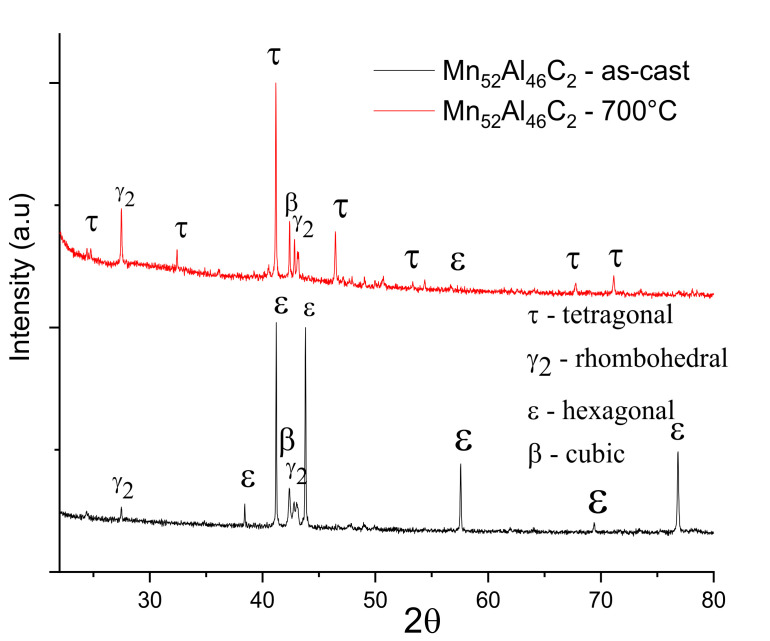
X-ray diffractograms for samples Mn_53_Al_45_C_2_ as-cast and annealed at 700 °C.

**Figure 4:**
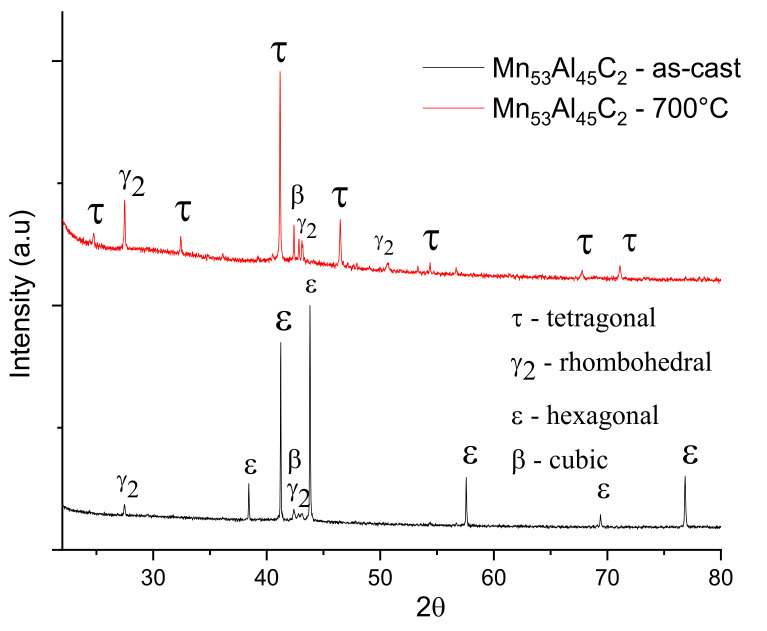
X-ray diffractograms for samples Mn_52_Al_46_C_2_ as cast and annealed at 700 °C.

The structural characterization of the as-cast and annealed alloys was done by X-ray diffraction at room temperature. XRD patterns have been recorded for both as-cast and annealed samples. Comparative XRD patterns are represented for Mn_53_Al_45_C_2_ and Mn_52_Al_46_C_2_ samples are shown in Figure 3 and Figure 4. For correct identification of the observed Bragg lines and to derive the needed lattice parameters for the crystalline phases occurring in the samples, a full-profile, Rietveld-type analysis was done with the MAUD software.

The Mn_53_Al_45_C_2_ *as-cast* sample exhibits polycrystalline behavior, having well-formed, sharp Bragg peaks, mostly assigned to hcp ε-phase. The full-profile analysis provided the lattice parameters of the *hcp* crystal structure: a = 2.684 Å and c = 4.336 Å. Based on the fitting parameters resulted from MAUD, peak angular positions and shape, full-width-at-half-maximum (FWHM), we estimated the grain size, understood here as being the volume weighted, crystallographically-coherent, domain size. We have thus obtained a mean grain size of around 185 ± 12 nm for the hcp ε-phase. Besides the formation of the ε-phase, which is predominant in the as-cast samples, we detected also the presence of γ_2_-rhombohedral and β cubic phases, in much smaller proportions. The Mn_53_Al_45_C_2_ sample annealed at 700 °C shows quite different features. The strong diminution, even disappearance of the Bragg peaks of ε-phase is observed, new diffraction lines of significant intensity appear, lines that are indexed as belonging to the τ-phase (space group P4/mmm, International Centre for Diffraction Data ICDD file 11-0520). Such lines have also been observed in [32] and indexed there also as belonging to the τ-phase. The relative proportion of the new τ-phase is very high, found to be around 88%. The remaining 12% abundance consists of rhombohedral γ_2_ phase and primitive cubic β-phase. Correlating this value with the disappearance of the Bragg lines of the hcp ε-phase, we can conclude that indeed, after the 700 °C annealing, the Mn_53_Al_45_C_2_ alloy undergo an almost full ε-τ structural phase transformation. For the whole picture of the phase composition after the 700 °C annealing, it have to be said that, besides the small remaining hcp ε-phase (space group P6/mmm ICDD file 00-048-1830), the full-profile fitting analysis allows identification of two other phases, i.e., rhombohedral γ_2_ phase (space group R3m (160) ICDD file 00-032-0021) and cubic β-phase (primitive cubic (space group P4_1_32), all with a total amount of 12%. The lattice parameters of the τ structural phase were found to be *a* = 2.747 ± 0.011 Å, *c* = 3.576 ± 0.008 Å. For the rhombohedral γ_2_, the lattice parameters are *a* = 12.543 ± 0.0041 Å, *c* = 15.723 ± 0.0021 Å, while for the hcp ε-phase, these are *a* = 2.691 ± 0.0031 Å, *c* = 4.356 ± 0.0033 Å. The average crystal size value of around 225 ± 12 nm was estimated using Scherrer method for the τ-phase. In the same way, a mean grain size of around 185 ± 9 nm was calculated for the ε-phase.

An almost similar situation is encountered also for the Mn_52_Al_46_C_2_ alloy, *as-cast* and also annealed at 700 °C (Figure 4). Here, the *as-cast* state is also characterized by a predominance of the hcp ε-phase which transforms after annealing in a more complex phase structure. In this case, the ε-τ structural phase transformation is also almost full, with a formed τ-phase of about 91%, almost complete disappearance of cubic β-phase, and the remaining 9% abundance being made of rhombohedral γ_2_ phase and primitive cubic β-phase, with lattice parameters almost equal to the previous case, within the range of estimation errors. The crystallite average size, calculated from the full profile analysis fitting results, shows almost similar values: 232 ± 14 nm for the two most intense lines of the τ phase in the annealed sample, and respectively 198 ± 15 nm for the ε phase in the *as-cast* sample.

To improve the accuracy of the estimations for the average grain sizes, also taking into account other contributions to the Bragg peak broadening, such as microstrain, we have also employed the integral breadth method [45,46]. This method uses parameters resulting from MAUD fitting, such as lattice parameters, line widths, and mixing parameters of each Bragg reflection, and calculates the average crystallographic domain size associated with the average grain size and the root mean square lattice microstrain. In this case, the lattice microstrain is defined as the amount (either in size or volume) by which a crystal lattice deforms under stress/strain and it is given as a ratio of the experimental lattice size to the initial theoretical lattice size, being a non-dimensional parameter [45]. During the non-equilibrium synthesis, the cooling of the ribbons occurs differently for the two sides of the ribbons, the one in contact with the Cu wheel and the other one cooling in the controlled atmosphere. Therefore, structural stress is expected to be induced during casting of the ribbons and, consequently, the lattice microstrains between the atomic planes could be significant. Such a parameter can be estimated using the integral breadth method. The results of our calculations for the average grain size of the τ-phase, as well as for the lattice microstrain, using the integral breadth method, are given in Table 1. The values of the average grain size are 244 ± 18 and 268 ± 25 nm, for the two samples Mn_53_Al_45_C_2_ and Mn_52_Al_46_C_2_, respectively. It can be seen that for both annealed samples the average grain size is consistently higher than the values obtained by the Scherrer method, with as much as 15%. The reason is that within the integral breadth method, the peak broadening as well as the asymmetry of the line profile is taken into account, together with the instrumental broadening of the Bruker D8 diffractometer and the microstrain contributions. Lattice microstrain determined from the fitting shows quite significant values that justifies discrepancies in the estimation of the average grain size, as discussed above and are to be correlated with the preferential texturing, explained hereafter.

In general, formation of crystallites during the rapid solidification from the melt or during annealing occurs with preferential spatial orientation. There are few theories trying to explain this preferential growth. The minimization of the surface energy and synthesis parameters were considered to produce preferential growth of crystals [47]. It has been shown [47,48] that, in the early stages of crystallization, surface energy dominates the preferential growth and the tendency is for *in-plane* growth anisotropy. With an increase in ribbons thickness, strain energy predominates and the growth may be preferential in the *out-of-plane* directions. Using MAUD results of the full-profile analysis, it is possible to estimate the preferential orientation of crystals of the τ-phase by investigating the texture coefficient, which can be calculated using the following formula [48]:

(1) $$T_{hkl}=n\left(\frac{I_{hkl}}{I_{hkl}^{0}}\right)\sum \frac{I_{hkl}}{I_{hkl}^{0}}$$

*T_hkl_* being the texture coefficient of the corresponding Bragg reflection, *I_hkl_* its integral intensity as determined by numerical fitting of XRD pattern, *I*^0^*_hkl_* corresponds to the bulk Bragg peak intensity (from the ICDD file), and n is the number of Bragg peaks observed. In Table 2, we present the texture coefficient results for the samples annealed at 700 °C for the three most prominent *(hkl)* reflections of the tetragonal τ-phase: (110), (111), and (200). It can be seen that while the texture coefficient of the main (111) peak of the tetragonal phase is slightly below 1, the two superlattice peaks (110) and (200) show both a texture coefficient which is larger than 1. This indicates a slight *in-plane* preferential orientation for the formation of the crystallites of the τ-phase.

We can conclude that in the case of ribbons in the as-cast state, the ε hexagonal phase predominates. Following the annealing at 700 °C, the ε phase has been almost completely transformed into the tetragonal τ phase which becomes predominant. The microstructure of the annealed samples is completed by rhombohedral γ_2_ and cubic β phases that coexist (small abundance) with the predominant τ phase.

### 3.3. Magnetic Properties

**Figure 5:**
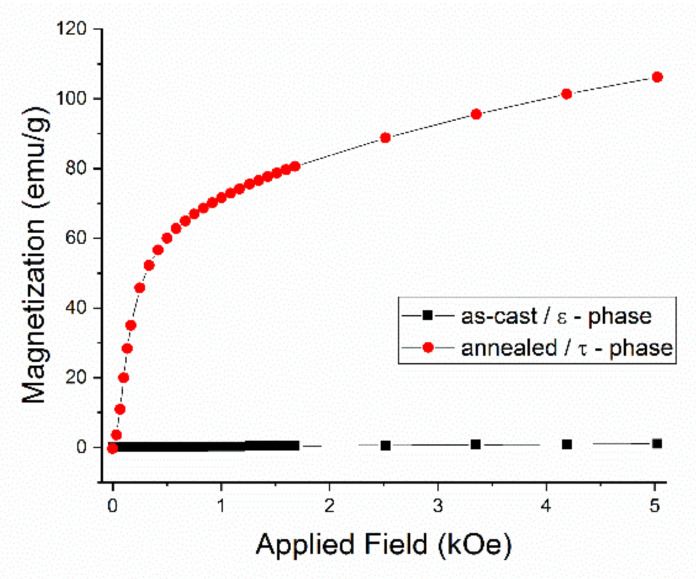
Initial magnetization for samples Mn_53_Al_45_C_2_ *as-cast* and annealed at 700 °C.

**Figure 6:**
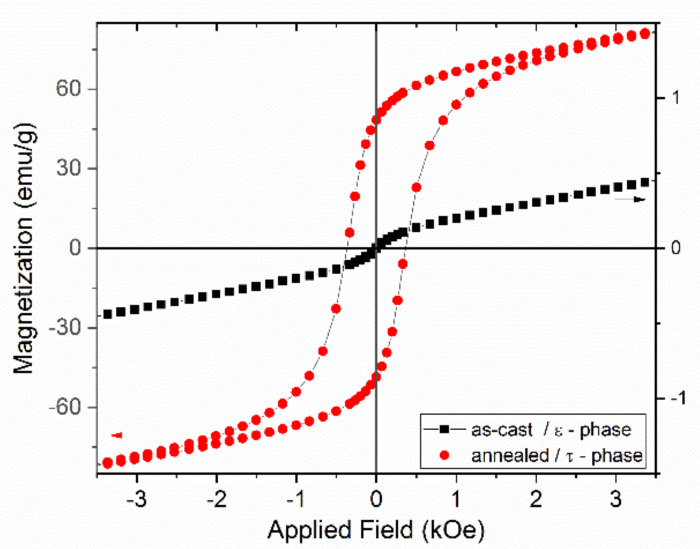
Hysteresis loops for samples Mn_53_Al_45_C_2_ as-cast and annealed at 700 °C.

The initial magnetization as well as the hysteresis loops of the *as-cast* and annealed Mn_53_Al_45_C_2_ have been measured using SQUID magnetometry in a measurement setup having the magnetic field applied parallel to the ribbons plane. The field is applied up to 80 kOe and data are recorded at 27 °C. The initial magnetization curves for the as-cast and annealed Mn_53_Al_45_C_2_ samples are shown in Figure 5 and the hysteresis loops of the same samples are depicted in Figure 6. The as-cast sample, where it has been shown that the hcp ε-phase is predominant, exhibits a very low, linear, increase of magnetization, a behavior which is typical for antiferromagnetic materials. The annealed sample, where it has been shown that the tetragonal τ phase has become predominant, have completely different features, with a sharp increase of magnetization towards high values (up to about 106 emu/g) and a steady approach to saturation. The ferromagnetic character assigned to the tetragonal τ phase lacks soft magnetic features, taking into account that the saturation is not reached even at the highest applied magnetic field (80 kOe). This behavior is preserved also for the recorded hysteresis loops (Figure 6). The as-cast sample has quite linear dependence on the applied magnetic field with very small induced magnetization, of the order of 1 emu/g (right Y axis on the graph). The annealed sample shows, in agreement with the initial magnetization behavior, very large values of magnetization, in addition to a quite important increase of the coercive field, up to about 0.4 kOe. The large value of the observed coercivity, viewed together with the lack of saturation of the measured values of magnetization are considered as being the consequence of an arrangement of frustrated moments of the Mn ions in the tetragonal τ phase.

These values obtained for the Mn-Al-C alloy with predominant τ phase are in agreement with previous reported values [16,32]. It is to be noted that the hysteresis loop of Mn_54_Al_46_ depicted by Fang et al. [38] has a very similar aspect with lack of saturation of magnetization and observable non-zero coercivity, but in our case the C addition has allowed a higher coercivity as well as a higher magnetization that leads to further increase the maximum energy product (BH)_max_, the figure of merit of any magnetic material. We already explained that the magnetic behavior of the *as-cast* sample is quite different. Whereas the hysteresis loop shows no coercivity, the increase of magnetization is steady and linear, showing no approach to saturation for magnetization of the sample. This suggests a pure antiferromagnetic character of the sample, a behavior that is imposed by the phase composition of the *as-cast* sample, where a predominance of hcp ε-phase has been detected from structural analysis. Such a shape of the magnetization and hysteresis loop was reported also by Janotova et al. [31] for the *as-quenched* Mn_55_Al_45_ alloys.

The results obtained on the Mn_53_Al_45_C_2_ annealed at 700 °C, i.e., a high coercive field of about 0.4 kOe, maximum magnetization of about 106 emu/g, and taking into account also the theoretical density of the Mn_53_Al_45_C_2_ of about 5.13 g/cm^3^, the upper limits of (BH)_max_ can be estimated at around 10.2 MGOe, in agreement with previous reported values [44].

## 4. Discussion

For many magnetic materials, there is a wide range of experimental evidence that different synthesis routes followed by appropriate annealing can give different and finely tuned intrinsic and extrinsic magnetic parameters [49,50,51,52,53,54,55,56,57,58]. It has been proven [59,60] that occurrence of magnetic hysteresis in Mn-Al alloys is most of the times associated with phenomena linked to domain wall pinning within stacking faults and also within antiphase boundaries. On the other hand, antiphase boundaries also have the role of imposing nucleation sites for the desired microstructure [61,62], and of creating distortions through local stress fields of dislocations [63]. Sometimes, the pinning mechanisms occurring at twin boundaries, found experimentally [64] and also theoretically [62], are deemed to impede both the coercivity and remanence.

Most of the times, Mn-Al and Mn-Al-based alloys fail to achieve high enough (BH)_max_. This is because the fraction of the obtained τ phase is low and because it is difficult to create a microstructure aligning the easy axis of this tetragonal phase. The τ phase usually forms from the hcp ε-phase; however, the typical approach of synthesis through ball milling leads to mixture of crystalline phases and an implicit decrease of the saturation magnetization [65,66,67,68,69]. In our case, the non-equilibrium synthesis procedure of fast solidification from the melt (at 1300 °C), combined with the compositional modulation induced by the presence of C atoms, have ensured a high enough fraction of τ phase. These gave rise to high saturation magnetization and large coercivities in our samples.

## 5. Conclusions

With the purpose of monitoring the ε-τ phase transformation and to maximize the effects of annealing in terms of increasing magnetic parameters, two melt spun alloys of compositions Mn_53_Al_45_C_2_ and Mn_52_Al_46_C_2_ have been synthesized through a non-equilibrium method, consisting of rapid quenching from high temperature of the molten state. It has been shown that the two samples exhibit different activation energies and different temperatures of onset of the ε-τ phase transformation. This difference is explained by the slight compositional changes that positioned one of the alloys closer to the limit of τ phase formation range, in the phase diagram, therefore requiring lesser activation energy for inception of the ε-τ transition. Structural analysis performed using XRD followed by full profile Rietveld-type analysis provided an appropriate image of the microstructure and phase composition of the alloys. Appropriate annealing was performed in order to refine the microstructure, enable the phase transformation, and realize suitable arrangements of magnetic regions capable of maximizing magnetic features. While hcp ε-phase was found to be predominant in the as-cast samples, after appropriate annealing, it was shown that conditions of annealing at 700 °C for 1 h are more than enough in order to achieve full ε-τ phase transformation. Thus, in the annealed samples, the tetragonal τ phase, responsible for the good magnetic properties, is found in overwhelming abundance of about 90%, the rest consisting a mixture of cubic β and rhombohedral γ_2_ structures. An image of hcp ε-phase that decomposes via atomic migrations at interphase and interfaces, having the transformation rate governed through boundary diffusion processes, may be constructed from our data. While for the as-cast sample Mn_53_Al_45_C_2_, consisting of predominant hcp ε-phase, antiferromagnetic behavior was proven, with very small values of magnetization and virtually no coercivity, the magnetic measurements of annealed sample Mn_53_Al_45_C_2_, consisting of predominant tetragonal τ-phase, provided high values of magnetization and increased coercivity, consistent with an energy product of about 10 MGOe, in agreement to values of magnetization reported in the literature and correlated to the structural characteristics and phases determined in Mn-Al-C alloys.

## Figures and Tables

**Table 1:** Lattice parameters, average crystal size of the τ-phase, and microstrain for the samples annealed at 700 °C. R_wp_ and χ^2^ (goodness of fit) are also provided.

Sample	Lattice Parameters			Size	Strain	R_wp_	χ^2^
	a (Å)	c (Å)	V (nm^3^)	(nm)	(%)		
Mn_53_Al_45_C_2_	2.747 ± 0.011	3.576 ± 0.008	0.0269	244 ± 18	0.89 ± 0.08	2.11	1.41
Mn_52_Al_46_C_2_	2.744 ± 0.013	3.572 ± 0.009	0.0268	268 ± 25	0.77 ± 0.09	2.05	1.24

**Table 2:** Texture coefficient results for the samples annealed at 700 °C.

Sample	Texture Coefficient		
	(110)	(111)	(200)
Mn_53_Al_45_C_2_	1.04	0.83	1.08
Mn_52_Al_46_C_2_	1.11	0.79	1.03

## Data Availability

The data are not publicly available due to patenting reasons.
